# Durable superhydrophobic/superoleophilic melamine foam based on biomass-derived porous carbon and multi-walled carbon nanotube for oil/water separation

**DOI:** 10.1038/s41598-023-31770-x

**Published:** 2023-03-18

**Authors:** Hadi Shayesteh, Mobin Safarzadeh Khosrowshahi, Hossein Mashhadimoslem, Farid Maleki, Yahya Rabbani, Hosein Banna Motejadded Emrooz

**Affiliations:** 1 Faculty of Chemical Engineering, Iran University of Science and Technology (IUST), Narmak, Tehran, 16846 Iran; 2 Nanotechnology Department, School of Advanced Technologies, Iran University of Science and Technology (IUST), Narmak, Tehran, 16846 Iran; 3 Department of Polymer Engineering and Color Technology, Amirkabir University of Technology, No. 424, Hafez St, Tehran, Iran; 4 School of Chemical Engineering, College of Engineering, University of Tehran (UT), Tehran, Iran

**Keywords:** Environmental sciences, Chemistry, Materials science, Nanoscience and technology

## Abstract

In the present study, fabrications of two eco-friendly superhydrophobic/superoleophilic recyclable foamy-based adsorbents for oil/water mixture separation were developed. Hierarchically biomass (celery)-derived porous carbon (PC) and multi-walled carbon nanotube (MWCNT) were firstly synthesized and loaded on pristine melamine foam (MF) by the simple dip-coating approach by combining silicone adhesive to create superhydrophobic/superoleophilic, recyclable, and reusable three-dimensional porous structure. The prepared samples have a large specific surface area of 240 m^2^/g (MWCNT), 1126 m^2^/g (PC), and good micro-mesoporous frameworks. The water contact angle (WCA) values of the as-prepared foams, PC/MF and MWCNT/MF, not only were 159.34° ± 1.9° and 156.42° ± 1.6°, respectively but also had oil contact angle (OCA) of equal to 0° for a wide range of oils and organic solvents. Therefore, PC/MF and MWCNT/MF exhibited superhydrophobicity and superoleophilicity properties, which can be considered effective adsorbents in oil/water mixture separations. In this context, superhydrophobic/superoleophilic prepared foams for kind of different oils and organic solvents were shown to have superior separation performance ranges of 54–143 g/g and 46–137 g/g for PC/MF and MWCNT/MF, respectively, suggesting a new effective porous material for separating oil spills. Also, outstanding recyclability and reusability of these structures in the ten adsorption-squeezing cycles indicated that the WCA and sorption capacity has not appreciably changed after soaking into acidic (pH = 2) and alkaline (pH = 12) as well as saline (3.5% NaCl) solutions. More importantly, the reusability and chemical durability of the superhydrophobic samples made them good opportunities for use in different harsh conditions for oil-spill cleanup.

## Introduction

Chemical discharges caused by wastewater containing organic solvents have led to the pollution of organic resources, severe ecological damage, and the loss of various species^1–6^. Numerous techniques for the removal and recovery of oils and organic solvents from water have attracted a lot of attention for a very long time. Commonly-used cleanup methods include adsorption, skimming, chemical dispersion, bioremediation, the use of chemical treatment agents, centrifugation, filtration, and in-situ burning methods classified into three main categories: physical, chemical, and biological^7–11^. These mentioned methods mainly have disadvantages such as transferring pollutants from one phase to another, high cost, low efficiency, time and energy consumption, and waste of human and material resources^12–14^. Skimming is one of the most commonly used methods, but it has a high cost, and the efficiency of separating oil from water is unsatisfactory. Therefore, the need to explore a highly efficient approach to separate oil from water is more important than ever.

The use of physical methods based on porous superhydrophobic/superoleophilic structures with high selectivity has been proposed as one of the most effective and straightforward high-efficiency separation methods for separating oily compounds from aqueous environments^15–17^. Two-dimensional and three-dimensional materials in various forms, such as fabrics, membranes, meshes, sponges, foams, and nanoparticles, can be used in porous structures to separate oils or organic solvents from water^10,18–23^. Two-dimensional porous substances, such as fabrics, membranes, and metal meshes, have a lower sorption capacity than three-dimensional porous materials, such as foams, sponges, and aerogels. Three-dimensional porous structures with unique wettability (superhydrophobic/superoleophilic or superhydrophilic/superoleophobic) can completely repel one phase and adsorb another phase due to high porosity, large surface area, and low density when exposed to water and oil mixture^24–27^. In addition, sponges and foams have good recyclability due to their elasticity, which is suitable for large-scale treatment of oily wastewater^28,29^. These superhydrophobic/superoleophilic structures are therefore more significant in the field of treating oily wastewater.

As commercial polymer foam, melamine foams are an excellent oil adsorbent due to the presence of a high amount of nitrogen in their structure and non-flammability^30–32^. But it should be noted that these structures naturally adsorb water and oil simultaneously. Therefore, using different modifiers or the deposition of superhydrophobic structures with low surface energy on the surface of foams can increase the hydrophobicity property of this material. As is well known, the two fundamental components of a superhydrophobic surface are low surface energy and micro/nanoscale rough features^33^. In recent years, extensive studies have been conducted to change the wettability of pristine melamine foams, especially the wettability of these structures. Researchers have used structures such as graphite^34^, magnetic nanoparticles^29^, SiO_2_^35,36^, graphene^26^, and cardanol-hexylamine-based benzoxazine^37^ to create roughness on the surface of melamine foam. Moreover, low surface energy materials such as fluoroalkyl silane^38,39^, N-dodecylthiol, PDA^35^, and octadecyltrichlorosilane^40^ have been used for modifying of the surface.

Nazhipkyzy et al. investigated the coating of hydrophobic soot on a melamine sponge surface for adsorbing oil products from water. They showed that the sorption capacity of the soot-coated melamine sponge was 24 g/g. Also, the reusable and recyclable properties of soot-coated sponges illustrated excellent sorption ability after 19 cycles toward petroleum oil^41^. In another work, silanized SiO_2_ microspheres were used as an additive to a melamine sponge to prepare a superhydrophobic/superoleophilic composite with a water contact angle of 153.2° and water sliding contact angle of 4.8° with a sorption capacity of up to 130 g/g by Zhang et al.^42^. Tan and Zhang synthesized trisiloxane-modified melamine sponges with a water contact angle of 139.3 with a sorption capacity of 52.9–140.1 times its weight^43^. Arumugam et al. used mono and di-functional benzoxazines to create micro/nanoscale rough structures on melamine foam for oil–water separation. They investigated the effect of the percentage amount of benzoxazines to change the hydrophilic nature of melamine to hydrophobic/oleophilic properties for separating some oils, such as soybean oil, mineral oil, and engine oil from the oil–water mixture^44^.

Although the conducted studies have obtained important results, they have deficiencies such as poor mechanical strength, sophisticated processes, and even a negative environmental impact^45^. Therefore, using environmentally friendly and low-cost strong superhydrophobic sponges is still necessary. Structures containing high amounts of carbon have been used in various processes^46–51^.

Herein, two carbon-based materials, a multi-walled carbon nanotube and hierarchically biomass (celery)-derived porous carbon synthesized using Fe–Ni/AC catalyst and green self-activation methods, respectively. The melamine foam was then loaded onto the surface and modified with polydimethylsiloxane using a conventional dip-coating technique to silanize it, producing a superhydrophobic melamine foam. The characterization of the samples was evaluated using N_2_ adsorption–desorption, Field emission scanning electron microscopy (FESEM), X-ray powder diffraction (XRD), Fourier transform infrared spectrometer (FTIR), and Energy dispersive X-ray spectroscopy (EDX). The WCA study was then completed to assess the particles' superhydrophobicity quantitatively. The sorption capacity of superhydrophobic samples was also examined for oil/water separation under various circumstances.

## Materials and methods

### Materials

Reagent-grade compounds of all types were utilized in this investigation without additional purification. The collection of waste celery was followed based on organizational and environmental regulations. The Environmental Association in Ray released permission for the collection of celery leaves. Celery waste was rinsed three times before self-activation to get rid of any obvious contaminants. The collection of celery-biomass was following the relevant institutional, national, and international guidelines and legislation. Permission for the plant sample collection was obtained from the Forest Association, Iran-Tehran. Hydrochloric acid (HCl 37%) was procured from Dr. Mojallali for the acid pickling process (Iran). To synthesize MWCNT, all mentioned materials were supplied by Dr. Mojallali Group, including nitric acid (HNO_3_ 55%), dichloromethane (CH_2_Cl_2_ 99%), and hydrochloric acid (HCl 37%) (Tehran, Iran). The Arman Energy Company purchased Ar (99.99%), acetylene (C_2_H_2_ 99.99%), and N_2_ (99.99%). Sigma-Aldrich supplied the Fe(NO_3_)_3_9H_2_O and Ni(NO_3_)_2_6H_2_O, while the Jacobi Company supplied the activated carbon (AC).

Pristine melamine foam was purchased with a diameter of 150–300 µm and a porosity of 99% from BAYERNTEX (Germany) without any treatment. Polydimethylsiloxane (PDMS) and its curing agents (Siligard 184) were supplied by Dow Corning Corporation. Olive oil, corn oil, and sesame oil were bought from a local store. Chloroform, ethyl acetate, dimethylformamide (DMF), hexane, and acetone were supplied from Merck Company, and silicone oil was bought from local vendors. Oil red (Bio Basic reagents) and methylene blue (Merck Co.) were applied for dyeing oils and water, respectively. Lab reagents such as NaOH, HCl, and NaCl were also supplied by Merck Company.

### Self-activating system for porous carbon (PC)

A sealed chamber, a tubular electric furnace, an air pump, and a condenser are all components of the self-activating system and are all linked by pipes. During the process of synthesis, no gas is emitted into the environment because the system is sealed. During the pyrolysis procedure, an aluminum boat with a particular quantity of biomass in it was placed in the hot zone. The gases created during the pyrolysis of the feeding biomass are circulated in a closed-loop channel with the aid of an air pump located inside the system. These gases aid in carrying out the activation process. Therefore, no further activating agent is needed. Along the course of the gases, a condenser is also built, which collects a part of the exhaust gases and condenses them into a liquid. In the initial phase of preparing porous carbons, gathered celery was dried at 80 °C for 24 h to eliminate moisture. After that, it was thoroughly crushed into fine powders using steel balls for 3 h in a ball mill to the appropriate micron size (400–700 µm). In the self-activating system, 10 g of the dried celery powder was immediately pyrolyzed at 700 °C with a heating rate of 5 °C/min and kept at final temperatures for 3 h. After cooling to ambient temperature, the resulting product was washed with 1 M HCl to eliminate any leftover contaminants, followed by deionized water until pH neutrality was achieved. The resultant porous carbon was finally dried at 85 °C for 12 h.

### Synthesis of MWCNT

**Figure 1:**
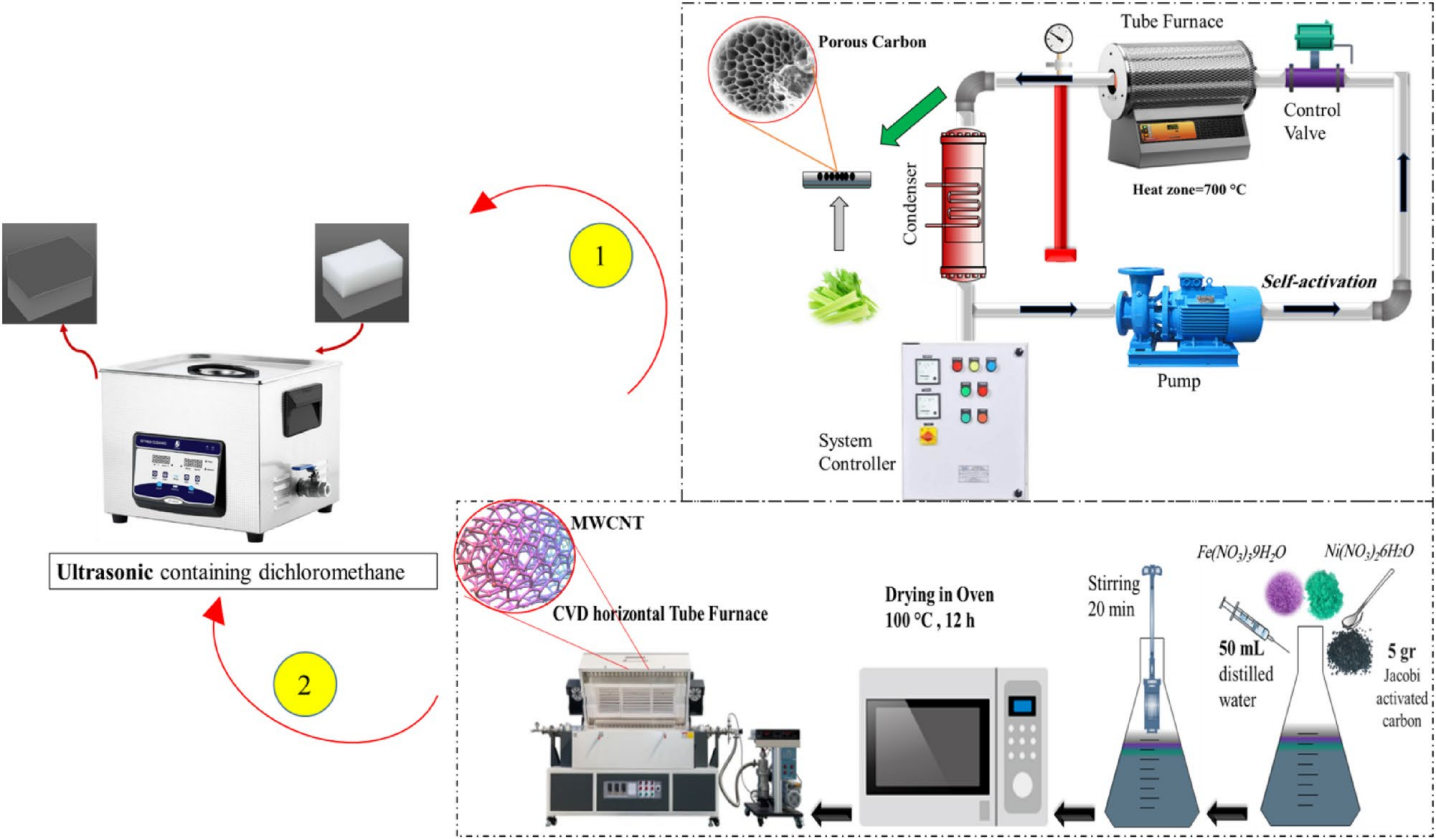
Process of carbon powders (PC and MWCNT) synthesis and impregnation of melamine foam.

The Fe–Ni/AC catalyst was prepared in a 200 mL conical flask containing 5 g of pure Jacobi activated carbon (AC), 50 mL of distilled water, and 0.25 M of Iron (III) Nitrate Nonahydrate and Nickel (II) Nitrate Hexahydrate. After reaching room temperature, the materials were crushed and sieved through a 200 µm sieve in a ball mill for 3 h under dry conditions using steel balls. The moisture and nitrates were then removed by heating the catalyst at 400 °C for 6 h. 5 g of the produced catalyst was placed in a ceramic boat affixed to the quartz tube (diameter 90 mm) of the CVD horizontal tube furnace. Through the passage of argon gas (30 mL/min) across the Fe–Ni/AC, system air is purged at a rate of 10 °C/min. The acetylene gas flow rate was set at 100 $mL/min$ for 30 min, while the argon gas flow rate was set at 250 mL/min at 700 °C. The acetylene flow was halted once the furnace reached room temperature, and the reactor was purged at a rate of 20 mL/min. The catalyst-produced MWCNT was extracted and sealed in a container, and its surface characteristics were assessed. Figure 1 is drawn to help explain the creation of porous carbon and MWCNT. This figure makes it evident that after being removed from the furnace, the synthesized powders are then moved to the ultrasonic device to initiate the impregnation and dispersing process.

### Preparation of the superhydrophobic/superoleophilic melamine foams

The melamine sponge was firstly cut into small pieces of 2 cm × 1 cm × 1 cm, following cleaned and washed twice with an ultrasonic using deionized water and ethanol for 30 min to achieve a clean sponge and then dried at 100 °C for 4 h in an oven. Then, 0.1 g of PC and MWCNT, 1 g of PDMS, and 0.1 g of curing agent were immersed in 25 mL dichloromethane. Subsequently, the clean MF (0.018 g) was added to the resulting suspension and sonicated for 1 h under N_2_ reflux in an ultrasonic bath. After that, treated MFs taken out and cured in a furnace for 2 h at 150 ℃ to get PC- modified MF (PC/MF) and MWCNT- modified MF (MWCNT/MF). Eventually, a pristine MF was submerged in PDMC for 0.5 h with sonication in order to more effectively compare the outcomes and examine the function of carbon powders.

### Oil/water separation process

Various oils and organic solvents, including chloroform, dichloromethane, silicone oil, olive oil, corn oil, sesame oil, ethyl acetate, toluene, dimethylformamide (DMF), hexane, and acetone, were chosen to show the ability of as-prepared foam to separate oil from water. Oils and water were firstly dyed with oil red and methylene blue, respectively to display them completely. Then, some oil was poured on the water. PC/MF and MWCNT/MF were immersed in oils and organic solvents to completely adsorb them. According to the mass of foams before (m_i_) and after (m_f_), the sorption capacity, (q (g/g)) can be determined as Eq. (1):

1 $$ q = \frac{{m_{i} - m_{f} }}{{m_{i} }} $$

### Characterization

Employing different characterization techniques, the characteristics of the created samples were examined qualitatively and quantitatively. The N_2_ isotherm models at 77 K were measured using a volumetric micro-politics ASAP2020 (Micromeritics Corp, USA) adsorption analyzer. Materials were autoclaved under cyclic vacuum pressure to constant mass at a temperature of about 155 °C for five hours before completing the adsorption–desorption analysis. Under a relative operating pressure of p/p_0_ = 0.055–0.20, the specific surface area was computed using the multipoint (BET) method, which means Brunauer–Emmett–Teller, as well as the total pore volumes, was measured at p/p_0_ = 0.955. The mesopore surface area, porosities, and pore size distribution could all be determined using the Barrett-Joyner-Halenda (BJH) method. The Dubinin-Astakhov (DA) approach is employed to compute the micropore size, whilst the t-method was utilized to estimate the micropore pore volumes and surface area (micropore). On a Perkin-Elmer Spectrometer, FTIR spectroscopy was carried out utilizing the potassium bromide (KBr) disc technique in the 500–4000 cm^−1^ band. On a Nanosem 450 microscope, FESEM was observed. A Philips (Holland) PW1730 diffractometer was used to acquire XRD spectra within 10 to 80° (2θ) using Cu-K radiation. The water contact angle (WCA) and oil contact angle (OCA) values were recorded using a digital optical microscope (DINOLITE, model AM-4113 ZT, Taiwan) at ambient temperature. The liquid drops (5 μL) were vertically placed on the surface of foams using a Hamilton microliter syringe. All contact angle were repeated at least three times at different locations and the average of results was reported. All liquid droplet images were processed by Image J® 1.51i software.

## Results and discussion

### Characterization

**Figure 2:**
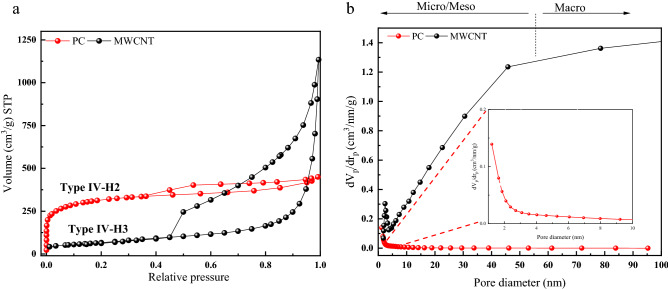
Nitrogen sorption/desorption isotherms at 77 K (**a**) and BJH pore size distribution profiles (inset is belonged to porous carbon) (**b**) of the porous carbon and MWCNT.

Nitrogen adsorption–desorption isotherms were used with a gas sorption analyzer to study the porosities and textural characteristics of prepared materials. Figure 2a, b and Table 1 evaluate the N_2_ adsorption–desorption analysis findings for the PC and MWCNT. The S_BET_, mean pore diameter, and total pore volume of the PC and MWCNT are 1126.2 m^2^/g, 2.5 nm, 0.69 cm^3^/g, and 240 m^2^/g, 14 nm, and 0.86 cm^3^/g, respectively. According to the BDDT (Brunauer, Deming, Teller, and L. S. Deming) classification, type IV, the typical curve for mesoporous materials can be seen^38,52^. The adsorbed volume of N_2_ increases with pressure for both C-based samples in the relative pressure range of 0.45 < P/P_0_ < 0.9 bar, which is consistent with adsorption on mesoporous/macroporous materials. When the relative pressure is greater than 0.9 bar, capillary condensation in the mesopores and macropores causes a rapid increase in the N_2_ sorption^53^. According to the original IUPAC categorization, PC exhibits H2-type hysteresis loops with typical columnar channels at relative pressures of 0.5–0.9 bar, whereas MWCNT exhibits H3-type (wedge-shaped pores)^54–56^. Based on BJH, the structure of PC hints at the presence of both micro and meso-porosity, whereas the structure of MWCNT indicates that the structure is entirely meso-structured^57,58^.

**Table 1:** Textural characteristics of the PC and MWCNT.

Sample ID	Total BET specific surface area (m^2^/g)	Average pore diameter (nm)	Total pore volume (cm^3^/g)	Mesoporous volume (cm^3^/g)	Microporous volume (cm^3^/g)
PC	1126.2	2.5	0.69	0.27	0.42
MWCNT	240.04	14	0.86	0.85	0.006

**Figure 3:**
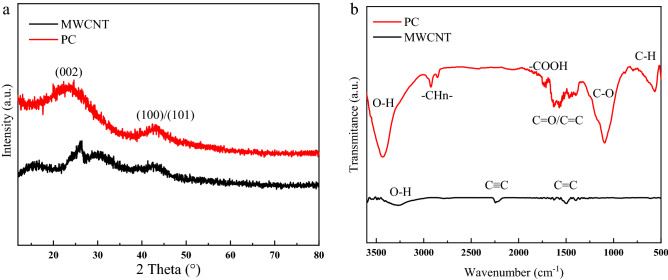
XRD patterns (**a**) and FTIR spectra (**b**) of the prepared porous carbon and MWCNT.

A multitude of variables such as active-site clustering, carbon framework, and inorganic contaminants influence the creation of pores. The internal structure of carbon is regarded to be the most important of these properties. The X-ray diffraction patterns exhibited in Fig. 3a illustrate the creation of carbon with a structural ordering intermediate between the amorphous-crystalline graphite phase^59–61^. The patterns of PC and MWCNT depict two broad Bragg reflections at 22–26° (002) and 42–44° (100/101), corresponding to the crystal planes of carbon^62–64^. The occurrence of these two peaks in PC validates the turbostratic structure (Poorly graphitized structure). This is a sign of the formation of amorphous-graphitic carbon during activation. For MWCNT, the first peak on the right indicates whether it is crystalline or amorphous; hence, the high width with low intensity, indicates that it exists in quasi-amorphous crystal form. The broadening of these peaks in both samples indicates a very tiny crystallite size and, as a result, the formation of a nanostructured skeleton^65,66^. Figure 3b depicts the surface functional groups of the synthesized carbons as determined by Fourier transform infrared (FTIR) spectroscopy. Peaks at ~ 3400 cm^−1^ can be found in both samples, which is compatible with the O–H stretching vibrations of hydroxyl groups^67–69^. The peak for PC is seen at about 2900 cm^−1^ and is attributed to asymmetric and symmetric − C–H_n_ methylene and methyl groups. For MWCNT, a minor peak at 2300 cm^−1^ can be seen, which corresponds to the C≡C band. Furthermore, the bond near 1700 cm^−1^ is linked to − COOH^70,71^. Stretching C=O bonds in carboxyl groups and C=C aromatic ring stretching vibrations create two bonds approximately 1500–1680 cm^−1^ in both prepared samples. The peak at 1100 cm^−1^ in the PC sample is caused by C–O bands in ether, phenol, and alcohol. In addition, the aromatic C–H vibration (600 cm^−1^) is detected in this sample^72,73^.

**Figure 4:**
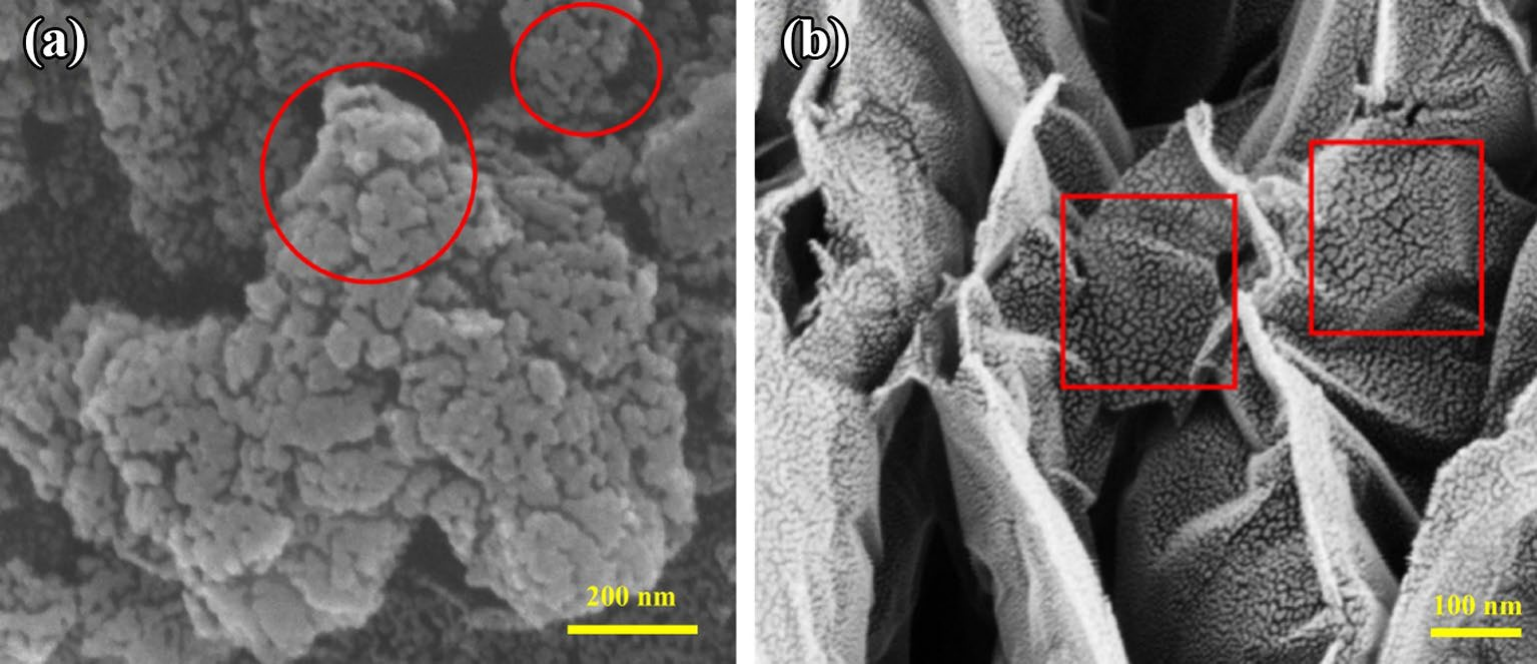
FESEM images of synthesized materials, (**a**) PC, (**b**) MWCNT.

With its visual examination of size, morphology, and skeleton, FESEM is a vital tool for assessing porous materials^74^. Figure 4a, b displays the micrographs of the PC and MWCNTs in their prepared state. In comparison to the MWCNT, the synthesized PC has a somewhat thicker and coarser morphology that results in the creation of a heterogeneous framework with many wrinkles^75,76^. Short and bent nanotubes that formed a porous network make up the synthesized MWCNT. The MWCNTs appear to be aggregating. Compared to PC, the nanotube surface's topography is less heterogeneous. The MWCNT also demonstrates the walls' smooth surface, random growth, homogenous diameter distribution, and a high degree of entanglement. It appears that the porous carbon's rough structure increases the air–water contact, which makes it easier for water droplets to dislocate easily throughout the surface^77–79^.

**Figure 5:**
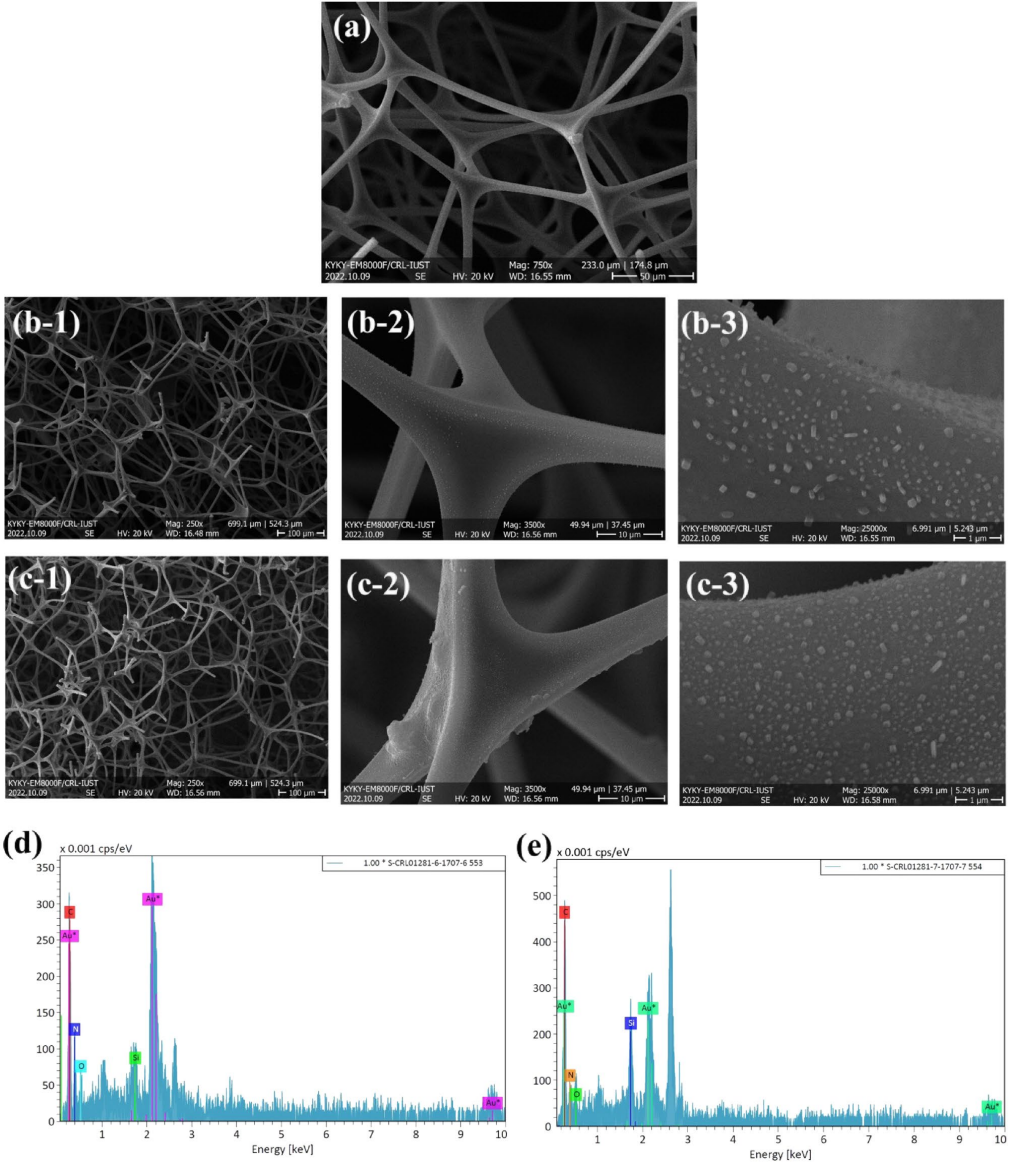
FESEM images of (**a**) pristine MF, (**b**) PC/MF, and (**c**) MWCNT/MF, and EDX spectrum of (**d**) MWCNT/MF and (**e**) PC/MF.

FESEM analysis was also applied to show the surface morphology of pristine MF, MWCNT/MF, and PC/MF (Fig. 5). It can be seen from Fig. 5a that the pristine MF has three-dimensional porous structures and indicate smooth surface. The pore size of pristine MF was measured as an equal 150–300 µm. The three-dimensional porous structure of MF naturally provides a high sorption capacity. After the deposition of PC and MWCNT on the surface of MF, the porous structure of MF remained (Fig. 5b, c), indicating that the grafting processes did not change the original structures of the MF. Of course, as clear from the images, by deposing particles on the surface of the MF, the surface has been completely covered and densely assembled on the MF cell walls, forming a micro-nano structure. As mentioned earlier, a micro/nanoscale rough structure is one of the main factors for making a superhydrophobic surface. The elemental compositions of MWCNT/MF and PC/MF are shown in Fig. 5d and e, respectively. Their main elements are C, O, N, and Si. The gold coating causes the visible Au peak. The presence of C and Si elements demonstrates that low surface energy compounds were created successfully during the dip-coating process. In general, the FESEM and EDX spectrum results showed that the use of PC and MWCNT with PDMS in the simple drop-coating process simultaneously achieved the two necessary factors for the construction of the superhydrophobic surface.

### Wettability properties

**Figure 6:**
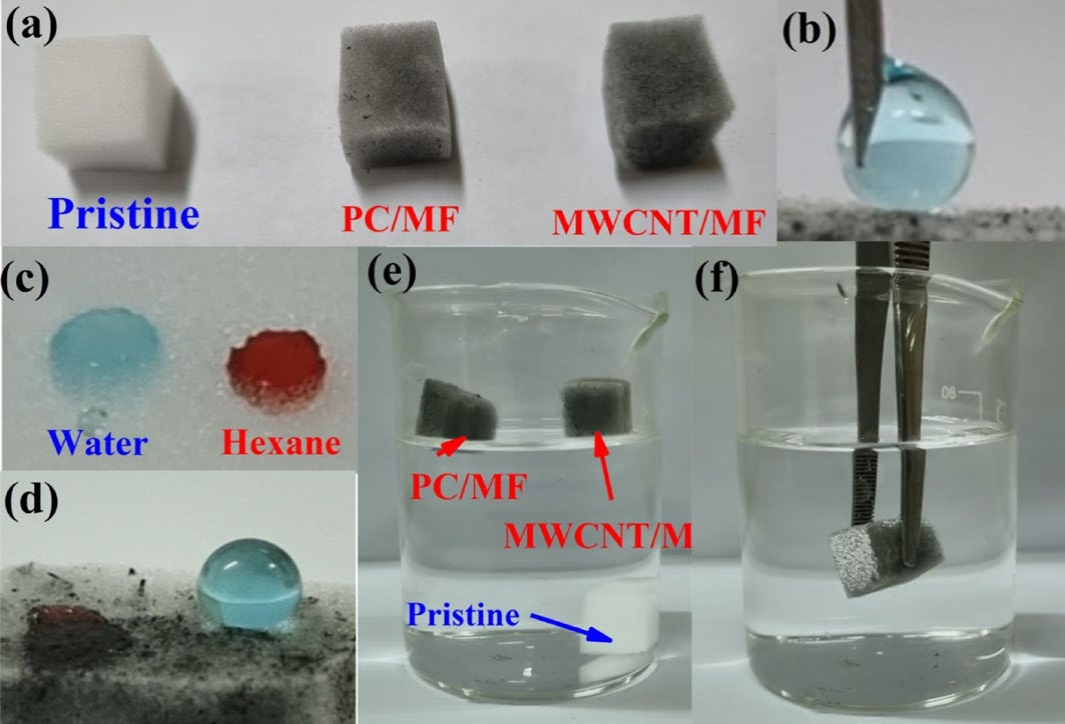
Digital photographs of (**a**) pristine MF, PC/MF, and MWCNT/MF, (**b**) the sessile water droplet method, (**c**–**e**) wettability behavior of MF and modified-MF, and the silver mirror-like interface by immerging of modified MF onto the water.

Figure 6a shows the difference in appearance between pristine MF, MWCNT/MF, and PC/MF. As it is known, the drop-coating method by using silicone adhesive Sylgard 184 has caused the particles completely covered the surface of three-dimensional porous structures so that the white color of the melamine foam has changed to black. This color change can be considered a reason for covering the particles on the surface of the foam. More importantly, the changes showed the wettability properties of the modified foams before and after the modification process. One of the most crucial factors to consider when assessing a material's wettability characteristics is the water contact angle (WCA) or oil contact angle (OCA). Hence, the sessile water droplet method with a volume of 5 µL was utilized so that the wettability of foams (MF, PC/MF, and MWCNT/MF) could be evaluated. Utilizing a Hamilton microliter syringe, vertical liquid droplets were put on the surface of the foam (Fig. 6b). Three contact angle measurements were carried out in various places, and the findings were provided according to their mean value^80^. As shown in Fig. 6c, pristine MF had a WCA and OCA equal to 5.1 and 0^°^, respectively. So, liquid (colored with methylene blue) and oil (colored with oil red) droplets quickly penetrated into the MF, which shows the simultaneous adsorption of water and oil. Differently, MWCNT/MF and PC/MF repelled water droplet and adsorbed oil droplets immediately, showing superhydrophobicity/superoleophilicity properties (Fig. 6d). As shown in Fig. 6e, the place of MF on the water caused them to sink into the water, but the modified foams remained on top of the water, following the superhydrophobicity property of MWCNT/MF, and PC/MF. The silver mirror-like interface was also indicated by immerging of modified MF onto the water (Fig. 6f). The trapping of air inside the three-dimensional structure of the foam has caused this phenomenon^34,81^.

**Figure 7:**
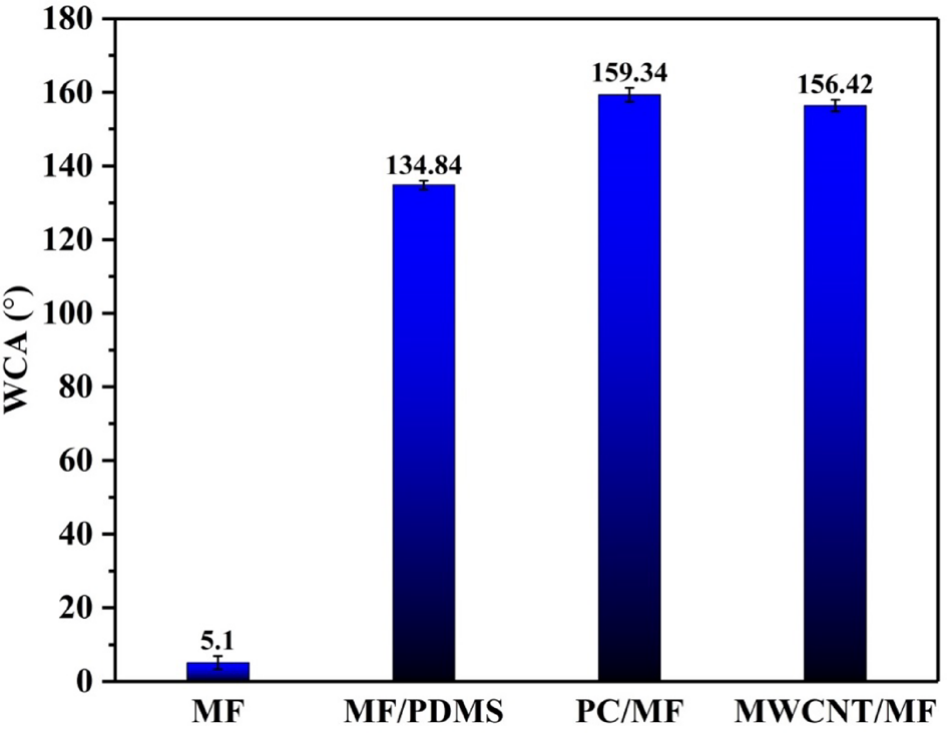
WCA variation of the liquid droplets on the pristine and modified MF.

Figure 7 depicts the WCA variation of the liquid droplets on the pristine and prepared foams. According to the findings, the WCA values for MF, MF/PDMS, PC/MF, and MWCNT/MF were 5.1° ± 1.8°, 134.84° ± 1.2°, 159.34° ± 1.9°, and 156.42° ± 1.6°, respectively. By assembling PC and MWCNT to MF and the subsequent use of silicone adhesive Sylgard 184, not only anchored powders on MF but also the hydrophilicity of MF has changed to hydrophobicity. The results of the modified foam with only PDMS and Sylgard 184 (MF/PDMS) show that the dip-coating has just created hydrophobic properties, but it still has an angle of less than 150°. Therefore, it is necessary to create roughness on the foam skeleton to achieve superhydrophobicity properties. In addition, it is essential to point out that the OCAs of a variety of oils were found to be equivalent to 0°. The findings demonstrate that both PC/MF and MWCNT/MF possess superhydrophobicity and superoleophilicity. Because of this, using these materials in processes that separate oil and water can be considered a viable option. The surface wettability of C-rich materials can be improved in terms of chemical characteristics by the large proportion of hydrophilic functional groups (C–O, C=O, etc.). On the other hand, physical characteristics including porosity, morphology, and surface roughness have a significant impact on hydrophobicity. The hydrophobicity of materials with rough surfaces can be increased because the porosity in these materials can capture air to create an "air pocket"^82^. According to FTIR results, surface oxygen species must provide a more polar and efficiently hydrophilic surface for porous carbon. But according to the results, it appears that the existence of more micropores and a rougher surface is more significant than the presence of functional groups. Also, according to EDX results, it seems that in the presence of PC, Si has played a more effective role in hydrophobicity (has a better connection to the surface of the MF). In other words, it is likely that MWCNT does not have a strong connection with the MF surface due to the existence of inherent strong bonds to prevent complete dispersion during sonication^83^.

### Oil sorption process

**Figure 8:**
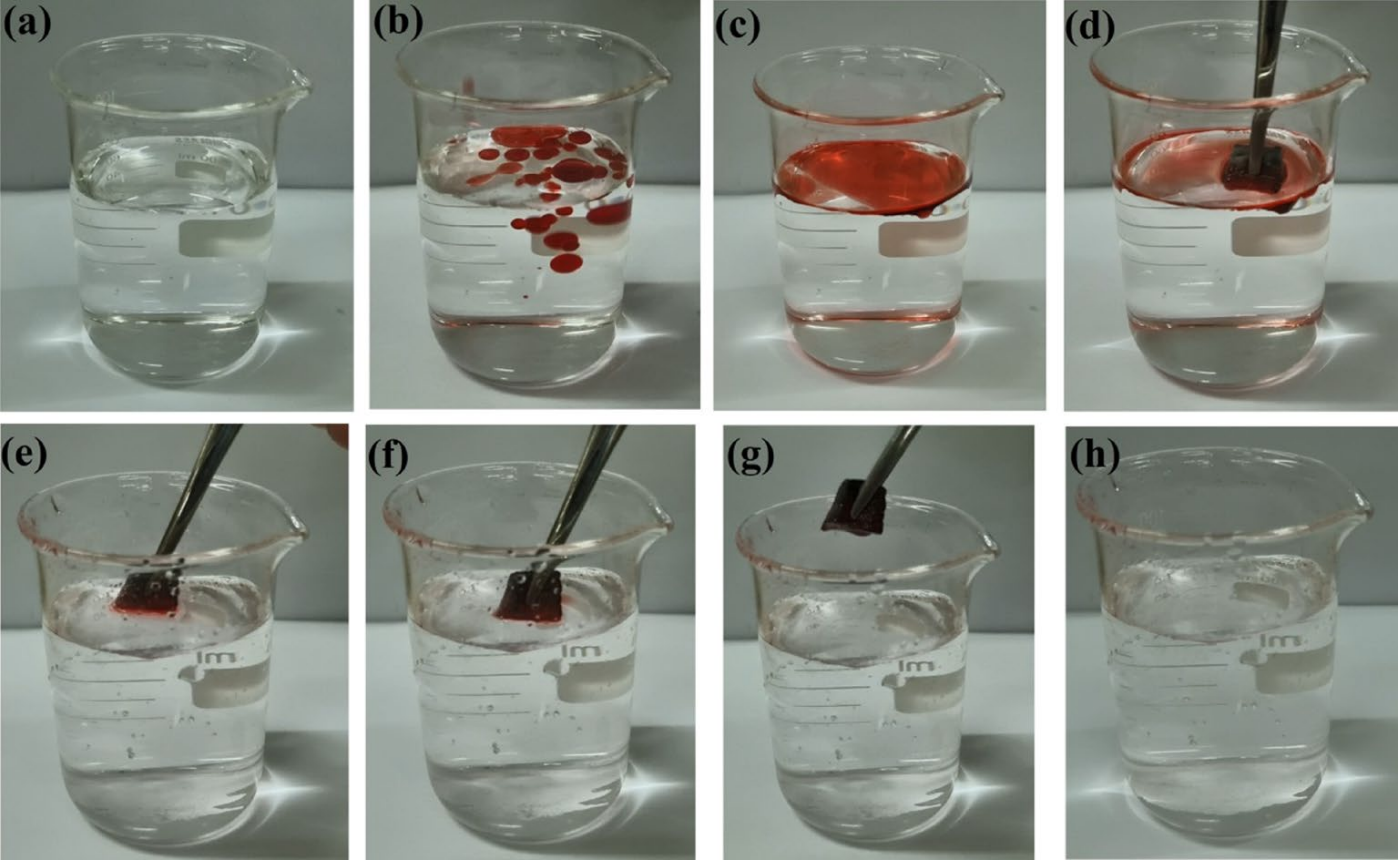
Digital photographs of oil spill absorbing floated on the water.

**Figure 9:**
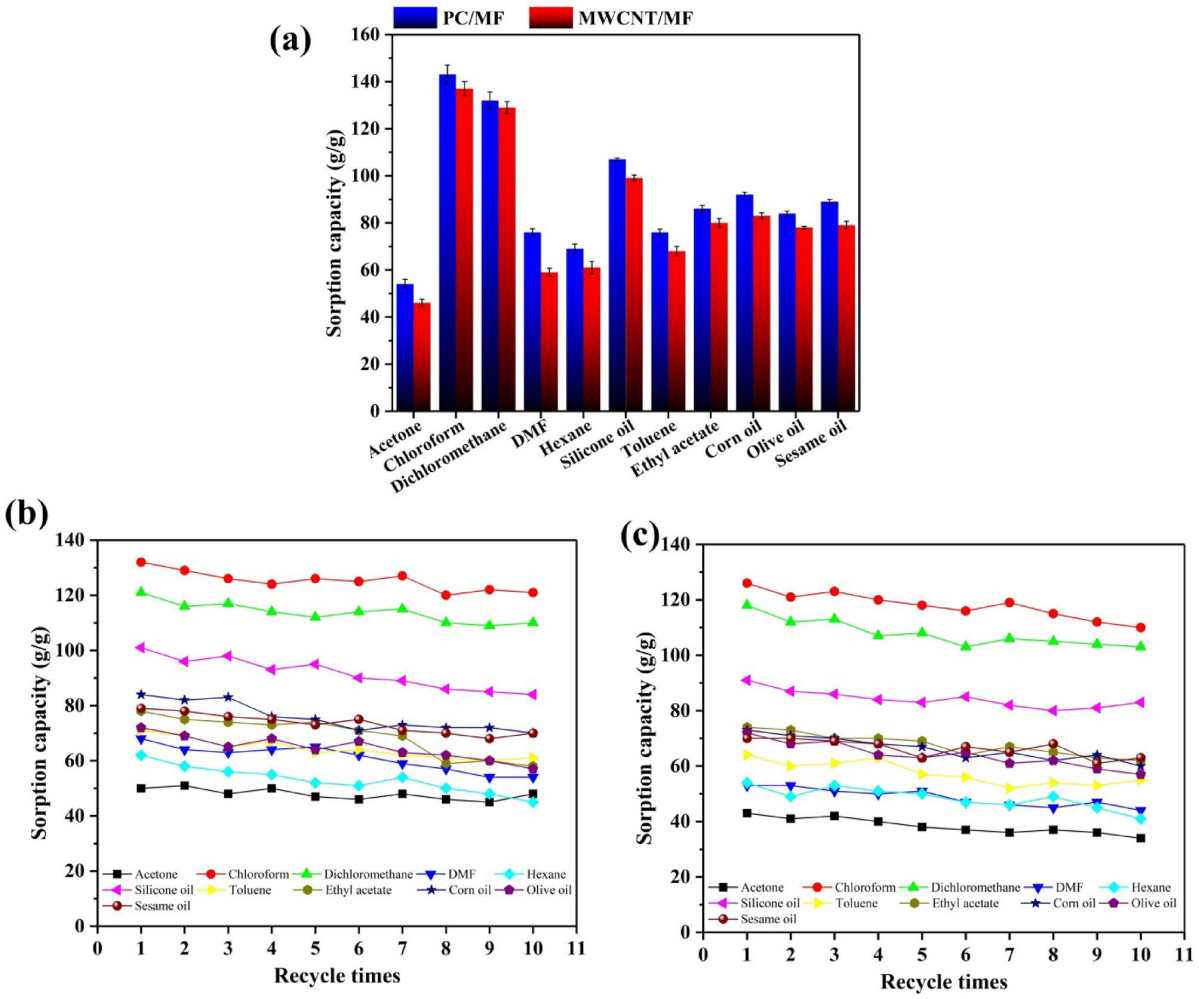
(**a**) The sorption capacity of PC/MF and MWCNT/MF and the sorption capacities of recyclable superhydrophobic/superoleophilic (**b**) PC/MF and (**c**) MWCNT/MF for selected oils and organic solvents in ten adsorption–desorption cycles.

Due to their unique superhydrophobicity/superoleophilicity qualities, PC/MF and MWCNT/MF can be considered very effective adsorbents in oil/water mixture separations. To further investigate the sorption capacity of as-prepared foams in oil/water mixture separation, eleven various model oils and organic solvents with different polarities, chloroform, dichloromethane, ethyl acetate, dimethylformamide (DMF), hexane, acetone, silicone oil, toluene, olive oil, corn oil, and sesame oil, were chosen. For example, in order to separate the light oils (*ρ*_oil_ < *ρ*_water_), oils and organic solvents were first dyed with oil red and subsequently sprinkled on top of the water. For separation of oils from the oil/water mixture in the batch system, the as-prepared modified foams were left on the oil spill and easily captured the oily target phase completely in about 3–5 min, following slick oil began to reduce at that point (Fig. 8a–h). Over time, the ultimate mass of the foams was weighed, and sorption ability was measured. As displayed in Fig. 9a, it could be seen that the measured sorption capacity for oils and organic solvents, including chloroform, dichloromethane, ethyl acetate, dimethylformamide (DMF), hexane, acetone, silicone oil, toluene, olive oil, corn oil, and sesame oil, were in the ranges of 54–143 g/g and 46–137 g/g for PC/MF and MWCNT/MF, respectively, which can compete with other used adsorbents (Table 2). The results showed that the highest and lowest sorption capacity belonged to acetone and chloroform, respectively.

**Table 2:** Sorption capacity of various melamine substrates.

Foam/sponge type	Oil/organic solvent sorption type	Water contact angle (°)	Sorption capacity (g/g)	Ref
Graphene/MS^1^	Diesel oil	–	99.0	^84^
Modified MF	Hexane, hexadecane, chloroform, dichloromethane, petroleum ether, gasoline, diesel, peanut oil, and lubricating oil	156	77–163	^37^
PVA^2^/PDMS/MF	Waste lubricating oil, dichloromethane, toluene, cyclohexane, petroleum ether, ethyl acetate, edible oil, acetone, atolin, and ethanol	157	46–85	^85^
Benzoxazines/MF	Engine oil, mineral oil, and soybean oil	158	85–95	^86^
Cellulose nanocrystals/MS	Hexadecane, dodecane, toluene, sunflower oil, styrene, dichloromethane, chloroform, cartbon, and disulfide	155	86–201	^45^
PDVB^3^ -PDMS/MS	Hexane, hexadecane, methanol, dichloromethane, acetone, colza oil, chloroform, pump oil, toluene, and DMF	160.9	38–123	^87^
PBZMS^4^/MS	Isooctane, gasoline, hexadecane, toluene, hexane, petroleum ether, and octane	164.2	62–170	^88^
PAF-HNTs-SiO_2_/MS	DMF, heptane, chloroform, dichloromethane, ethanol, toluene, acetone, hexane, methanol, and tetrahydrofuran	158	69–163	^89^
TDA^5^-MXene /MS	Cyclohexane, lubricating oil, petroleum ether, toluene, silicone oil, soybean oil, hexane, and diesel oil	> 150	60–112	^90^
Silanized SiO_2_/MS	Colza oil, soya-bean oil, engine oil, methylbenzene, cyclohexane, diesel oil, dichloromethane, gasoline, and kerosene	153	70–130	^91^
PCL-PDLLA^6^/MS	Hexane, octane, soybean oil, crude oil, and motor oil	162	3–8	^43^
PC/MF	Chloroform, dichloromethane, ethyl acetate, DMF, hexane, acetone, silicone oil, toluene, olive oil, corn oil, and sesame oil	159	54–143	Present study
MWCNT/MF	Chloroform, dichloromethane, ethyl acetate, DMF, hexane, acetone, silicone oil, toluene, olive oil, corn oil, and sesame oil	156	46–137	Present study

^1^Melamine sponge.

^2^Polyvinyl alcohol.

^3^ Polydivinylbenzene.

^4^ Polybenzoxazine.

^5^Tetradecylamine.

^6^Polycaprolactone/Poly-D, L-Lactic acid.

One of the most important factors of different porous adsorbents is the recyclability and reusability of these structures in the several adsorption–desorption cycles. After each test, the oils adsorbed by the foams were taken out by manual squeezing and a vacuum pump and used in the next sorption cycle. The sorption capacities of recyclable superhydrophobic/superoleophilic PC/MF and MWCNT/MF for selected oils and organic solvents are presented in Fig. 9b and c, respectively, which indicates that the sorption capacity has not appreciably changed even after 10 separation cycles. For instance, the sorption capacity of chloroform revealed that after ten cycles, the original sorption capacity was reduced by just 11 g/g and 16 g/g for PC/MF and MWCNT/MF, respectively.

### Chemical stability

**Figure 10:**
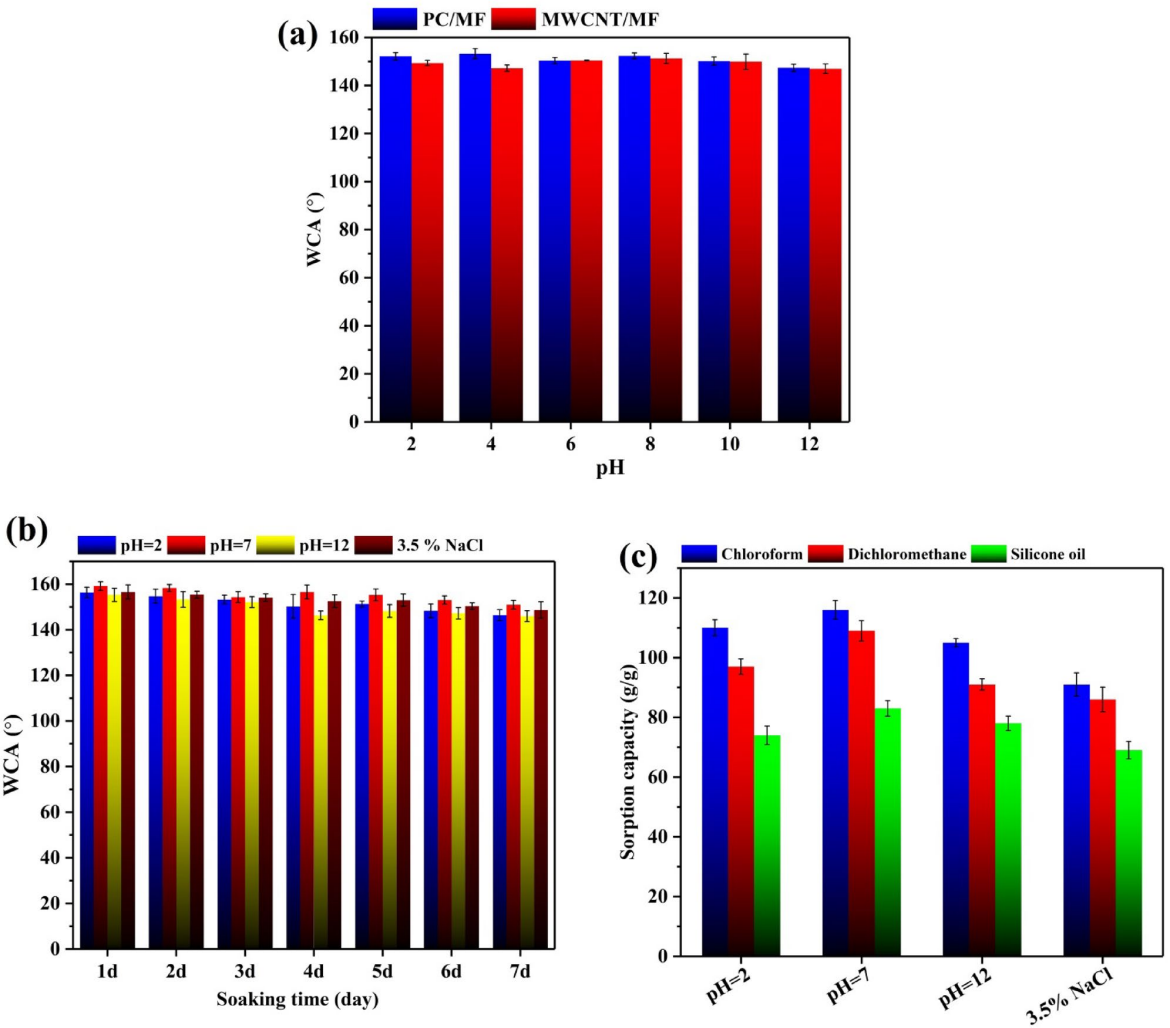
(**a**) Contact angle of droplets with different pH on PC/MF and MWCNT/MF, (**b**) WCA and (**c**) sorption capacity of MWCNT/MF in acidic, alkaline, and saline environments.

Wetting behavior and chemical stability under a wide range of adverse environments (acidic, alkaline, and saline solution) is the main problem with superhydrophobic/superoleophilic surfaces to apply in practical applications. In this study, the effect of the droplet with various pH on the wettability property of as-prepared foams was first investigated. As illustrated in Fig. 10a, the changes in the WCA of droplets with pH from 2 to 12 have been very insignificant, so the difference between the lowest and the highest WCA is less than ~ 7^°^. Furthermore, MWCNT/MF was also used to examine the effects of soaking times (one week) into acidic (pH = 2), water, alkaline (pH = 12), and saline (3.5 wt% NaCl) solutions on WCA variations and sorption capacity. The results showed that the selected foam could meet practical needs in severe and hard settings since its sorption capacity for oils remains roughly constant even in acidic, alkaline, and high-saline environments (Fig. 10b, c). Superhydrophobic samples are shown to have outstanding chemical durability and physical stability in steady or flowing settings, making them a prime candidate for oil/water separation technology.

### Mechanical strength

**Figure 11:**
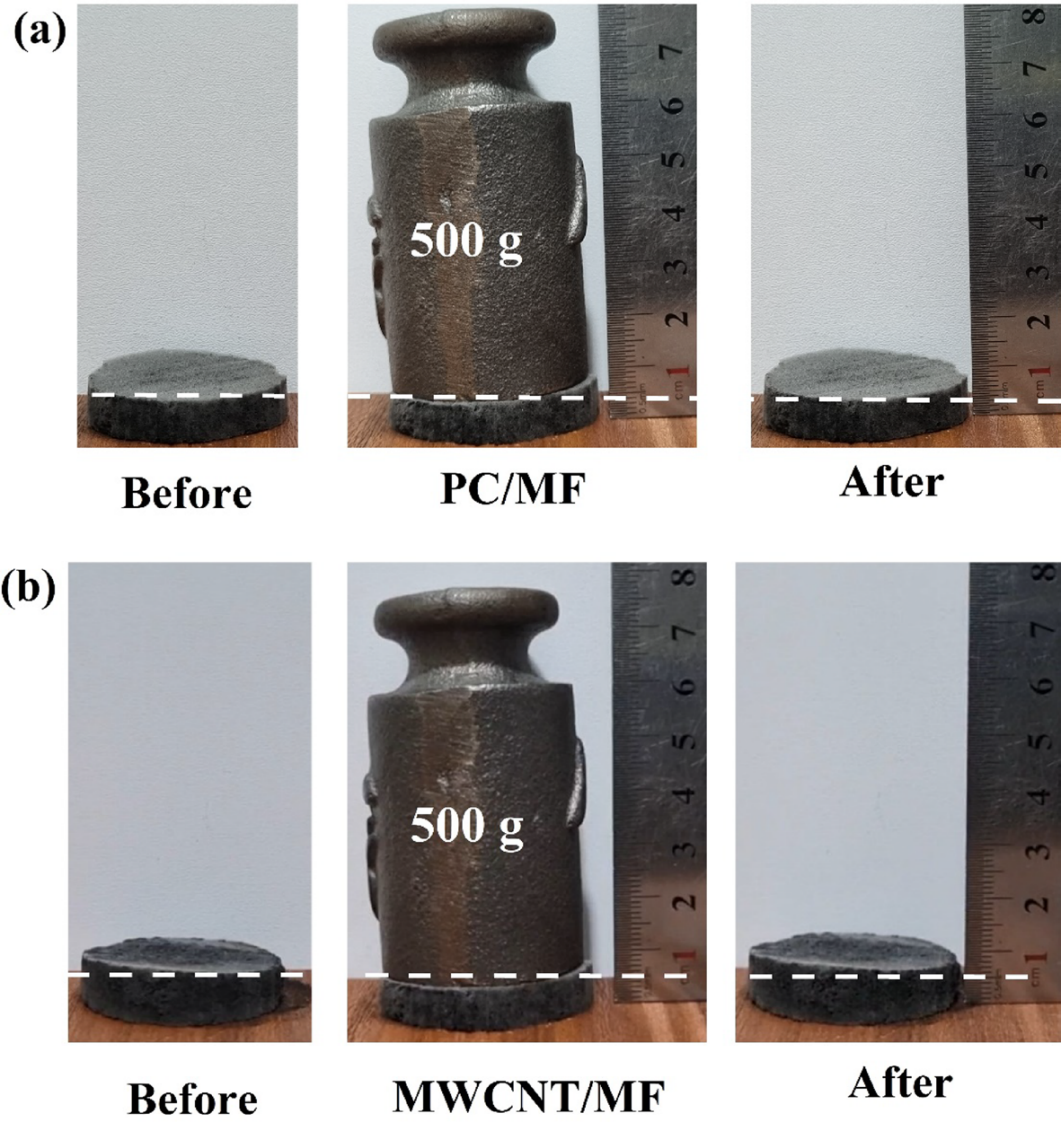
Mechanical strength test images of (**a**) the PC/MF and (**b**) MWCNT/MF before and after loading a counterweight.

Mechanical strength is one of the important parameters for practical applications in oil/water separations. Figure 11 shows PC/MF and MWCNT/MF could tolerate 31.38 kPa (the volume of prepared foams was 1.25 cm^3^) pressure without any deformation after loading a counterweight. The mechanical test suggested that the PC/MF and MWCNT/MF were not compressible which can be useful in various media.

## Conclusion

In conclusion, durable, reusable as well as recyclable superhydrophobic/superoleophilic melamine foams based on hierarchically biomass-derived porous carbon and multi-walled carbon nanotubes were prepared through a simple dip-coating route to apply in oil/water mixture separation. The presence of appropriate porosity in different dimensions, inherent hydrophobicity, easy synthesis and the heterogeneity of the surface of these two prepared samples have led to their use for the separation process. The WCA of 159.34° ± 1.9° and 156.42° ± 1.6° were recorded for PC/MF and MWCNT/MF, respectively, showing superhydrophobic/superoleophilic properties. The measured sorption capacities for various oils and organic solvents including chloroform, dichloromethane, ethyl acetate, dimethylformamide (DMF), hexane, acetone, silicone oil, toluene, olive oil, corn oil, and sesame oil, were in the ranges of 54–143 g/g and 46–137 g/g for PC/MF and MWCNT/MF, respectively. Also, the sorption capacities of recyclable superhydrophobic/superoleophilic foams indicated good recyclability and reusability even after ten adsorption-squeezing cycles. Besides, superhydrophobic surfaces have outstanding chemical durability and physical stability in acidic, alkaline, and high-saline circumstances, making them a prime candidate for oil/water separation technology. According to the findings, the selected foams is a promising sorbent for applications in the cleanup of oil spills and organic solvents from the aqueous environment. Besides, according to the results, the porous carbon obtained from biomass with the green self-activation method can be a suitable substitute for an expensive material such as MWCNTs.

## Data Availability

All data generated or analyzed data for experimental part during this study are included in this published article. Moreover, all other data that support the plots within this paper and other findings of this study are available from the corresponding author upon reasonable request. If you need to find out about the data, you can contact the following email: m_safarzadeh@nt.iust.ac.ir.
